# Editorial: The Effect of Business Cycles on Population Health in the Emerging Economies, Volume II

**DOI:** 10.3389/fpubh.2021.812976

**Published:** 2021-12-15

**Authors:** Wen-Yi Chen

**Affiliations:** Department of Senior Citizen Service Management, National Taichung University of Science and Technology, Taichung, Taiwan

**Keywords:** business cycles, population health, emerging economies, economic growth, economic development

Since their influential study on the relationship between business cycles and social conditions, Ogburn and Thomas demonstrated the significant association of economic expansion with increases in mortality in the early of 1920s (1). Their results were counter-intuitive from the classic economic growth theory (2) and raised significant arguments regarding whether or not the income view and health view hypotheses underpinning the positive effect of business cycles on population health can be validated in the real-world economy (3–5). Despite a changing relationship between business cycles on population health was usually found in the literature (6, 7), most evidence from previous studies was based on advanced economies. In this Research Topic, we aim to collect empirical results generated by different methodologies and various concepts of population health and economic development used to identify the relationship between business cycles and population health in the emerging economies.

The studies collected in this volume could be divided into three categories: the first being associated with the income view hypothesis, suggesting economic expansion (recession) boosts (reduces) accumulation of human capital, and thereby further improves (deteriorates) population health, the second being linked with the income view hypothesis, recommending that population health has a directly impact on income through human capital, and the third being the miscellaneous Research Topics that are related to target variables such as population health and economic development (a board concept of business cycles or economic fluctuation) under investigation of this volume.

The first category of this volume addresses the effect of business cycles on population health. In this category, many researchers applied different economic development indicators (such as GDP per capita, unemployment rate, urbanization rate, development of digital economics, technological innovation, industrial agglomeration, and participation in the social pension scheme) to measure business cycles (or economic fluctuation) and various measurements for population health (such as mortality rate, prevalence rate of depression and malnutrition rate of children under 5 years old) were also used to investigate the business cycles-population health nexus. According to their empirical results, the relationship between business cycles and population health was identified to be *positive* (Chai, Li et al.; Hu and Yao; Jiang et al.; Li S.-J. et al.; Pan et al.; Sun et al.; You et al.), *negative* (Zhong et al.), and *mixed* (Chai, Yang, Cui et al.; Su C.-W. et al.). Since the healthcare expenditure and healthcare insurance are frequently used to measure health capital formation and investment in health that are essential elements to economic growth (2, 3), several researchers investigated the impact of economic fluctuation (measured by GDP per capita, economic policy uncertainly, industrial air pollution, high-speed rail coverage, energy consumption, and development of insurance industry) on healthcare expenditure and health insurance. The positive association between economic fluctuation and healthcare expenditure was verified by Bai et al. and Niu et al. Nevertheless, Song et al., Pu et al., and Shen et al. found a changing relationship between economic fluctuation and healthcare expenditure. In addition, the positive effects of energy consumption and development of insurance industry on health insurance were justified by Wang K.-H. et al. and Li, Wang et al., respectively.

The second category of this volume emphasizes the impact of health capital formation (such as healthcare expenditure and health insurance) on economic activities or economic outcomes (such as economic growth, consumption, poverty, participation in financial market, and change of financial asset allocation). The effect of health capital formation on economic outcomes was identified to be *positive* (Cheng et al.; Wang Q.-S. et al.; Zhao et al.) and *ambiguous* (Jiang; Wu et al.). Moreover, some studies such as Li and Yang and Shi et al. demonstrated a significant influence of medical insurance on financial asset allocation and stock market participation, and other research illustrated that the outbreaks of pandemic diseases such as the COVID-19 and other infectious diseases significantly change the investor's behaviors (Fang et al.; Liu et al.; Nian et al.; Zhang, Zhu et al.).

The third category of this volume is miscellaneous Research Topic consisting of the evaluation of the efficiency of healthcare utilization, healthcare care and health insurance projects (Chai, Yang, Xie et al.; Chu et al.; Zhang, Guan et al.), environmental protection-governance relation (Hsiao et al.; Chang et al.), convergence of healthcare expenditure across different countries (Li Z.-Z. et al.), effect of health behavior on the individual's health (Li, Chen et al.), occupational health (Yao and Tao), firm performance in the pharmaceutical industry (Su C.-Y. et al.), healthcare reform (Luca et al.) and among others.

In sum, the studies collected in this volume contribute the literature focusing on the business cycles-population health nexus from three aspects: First, these studies applied the most sophisticated methodologies that provide the solid evidence on the relationship between business cycles and population health in the emerging economies. The research methodologies adopted by the studies collected in this volume include the Ability-To-Pay Approach, Categorical Variable Regression Analysis, Cluster Analysis, Data Envelopment Analysis, Difference-in-Differences Method, Panel Data Analysis, Social Network Analysis, Spatial Econometric Analysis, Structural Equation Modeling, and Time Series Analysis. Second, the extensive concepts of business cycles (namely, economic development) and population health were used to investigate the connection between economic development and population health (or health capital formation). Third, the findings generated from these studies in the first and second categories of this volume demonstrated the ambiguous results of the business cycles-population health relationship in the emerging economies. Namely, the income view and health view hypotheses based on the classic economic growth theory (2, 3) are not necessarily validated in the emerging economies. These results are consistent with previous studies on the relationship between business cycles and population health in advanced economies (4–7). Finally, it is important to address that bi-directional causation between income and health is a key feature of the classic economic growth theory that derives the income view and health view hypotheses (2, 3), but few studies in this volume formally deal with this issue. Therefore, we expect more research would be conducted to deal with the endogeneity between income and health in order to provide more sufficient evidence for the linkage of business cycles and population health in the future.

## Author Contributions

The author confirms being the sole contributor of this work and has approved it for publication.

## Conflict of Interest

The author declares that the research was conducted in the absence of any commercial or financial relationships that could be construed as a potential conflict of interest.

## Publisher's Note

All claims expressed in this article are solely those of the authors and do not necessarily represent those of their affiliated organizations, or those of the publisher, the editors and the reviewers. Any product that may be evaluated in this article, or claim that may be made by its manufacturer, is not guaranteed or endorsed by the publisher.
